# HIV self-testing acceptability among injured persons seeking emergency care in Nairobi, Kenya

**DOI:** 10.1080/16549716.2022.2157540

**Published:** 2023-01-11

**Authors:** Adam R. Aluisio, Scarlett J. Bergam, Janet Sugut, John Kinuthia, Rose Bosire, Eric Ochola, Beatrice Ngila, Kate M. Guthrie, Tao Liu, Mary Mugambi, David A. Katz, Carey Farquhar, Michael J. Mello

**Affiliations:** a Department of Emergency Medicine, Alpert Medical School of Brown University, Providence, RI, USA; b Department of Accident and Emergency, Kenyatta National Hospital, Nairobi, Kenya; c Department of Research & Programs, Kenyatta National Hospital, Nairobi, Kenya; d Center for Public Health Research, Kenya Medical Research Institute, Nairobi, Kenya; e Department of Psychiatry and Human Behavior, Alpert Medical School, Brown University, Providence, RI, USA; f Department of Biostatistics, Center for Statistical Sciences, Brown University School of Public Health, Providence, RI, USA; g Ministry of Health, Nairobi, Kenya; h Department of Global Health, University of Washington, Seattle, WA, USA; i Department of Epidemiology, University of Washington, Seattle, WA, USA; j Department of Medicine, University of Washington, Seattle, WA, USA

**Keywords:** Injury, HIV self-testing, Kenya, emergency medicine, HIV prevention

## Abstract

**Background:**

Emergency department-based HIV self-testing (ED-HIVST) could increase HIV-testing services to high-risk, under-reached populations.

**Objectives:**

This study sought to understand the injury patient acceptability of ED-HIVST.

**Methods:**

Injury patients presenting to the Kenyatta National Hospital Accident and Emergency Department were enrolled from March to May 2021. Likert item data on HIVST assessing domains of general acceptability, personal acceptability, and acceptability to distribute to social and/or sexual networks were collected. Ordinal regression was performed yielding adjusted odds ratios (aOR) to identify characteristics associated with high HIVST acceptability across domains.

**Results:**

Of 600 participants, 88.7% were male, and the median age was 29. Half reported having primary care providers (PCPs) and 86.2% reported prior HIV testing. For each Likert item, an average of 63.5% of the participants reported they ‘Agree Completely’ with positive statements about ED-HIVST in general, for themselves, and for others. In adjusted analysis for general acceptability, those <25 (aOR = 1.67, 95%CI:1.36–2.08) and with prior HIV testing (aOR = 1.68, 95%CI:1.27–2.21) had greater odds of agreeing completely. For personal acceptability, those with a PCP (aOR = 3.31, 95%CI:2.72–4.03) and prior HIV testing (aOR = 1.83, 95%CI:1.41–2.38) had greater odds of agreeing completely. For distribution acceptability, participants with a PCP (aOR = 2.42, 95%CI:2.01–2.92) and prior HIV testing (aOR = 1.79, 95%CI: 1.38–2.33) had greater odds of agreeing completely.

**Conclusions:**

ED-HIVST is perceived as highly acceptable, and young people with prior testing and PCPs had significantly greater favourability. These data provide a foundation for ED-HIVST programme development in Kenya.

## Introduction

Globally, it is estimated that one in eight people living with HIV (PLHIV) are unaware of their status [1]. In Kenya, young people contribute to over half of the national HIV incidence, with fewer men than women seeking conventional HIV testing services (HTS) [2]. There continues to be a need to improve HTS delivery to under-tested groups using acceptable and feasible approaches [3,4].

HIV self-testing (HIVST) is an innovative approach, endorsed by the World Health Organisation (WHO), to increase HIV testing in low- and middle-income countries (LMICs) [5,6]. HIVST can empower individuals to decide the setting and circumstances by which they test [7]. HIVST has been found to be feasible in African countries, such as Kenya [8]. Two studies from Zimbabwe and South Africa have shown that young adults, in particular, have demonstrated high uptake and favourable experiences with HIVST [9,10]. Additionally, a systematic review of data drawn from 13 countries in Africa found that men have a desire for HIVST access and use [11,12]. The Kenyan Ministry of Health (MOH) supports HIVST distribution in both the private and public sector, particularly for groups that have not been well engaged with standard HTS approaches [13].

Emergency departments (EDs) are an underutilized point of contact for many individuals who may also have high risk for HIV acquisition [14]. The Centers for Disease Control and Prevention (CDC) have identified EDs as key settings for HIV testing and linkage to care globally [15]. Data from LMIC demonstrate a median rate of approximately 30,000 visitations per year per emergency care facility, with the majority being men seeking care for injuries [16]. This profile suggests that EDs in LMICs may be impactful venues to leverage HIVST programmes as they interface predominately with younger men who, which in Africa have been under-reached for HTS. Additionally prior work in Western Kenya has found the HIV-positivity rate to be 22.7% in an ED setting [17]. A 2021 study from Nairobi of ED-based HIV-testing found a prevalence of infection of 11.4% among a cohort of predominately male-injured patients, a prevalence of disease twofold higher than the general population, illustrating the high-risk HIV profile among the ED patients [18]. The provision of HIVST kits from EDs in high-income settings has been shown to be acceptable to patients and successful in increasing HIV testing but data from populations in Africa at high risk for HIV have not been established [6,19]. To address that gap in evidence, the current study evaluated the acceptability of HIVST programmes among persons with injuries seeking emergency care in Nairobi, Kenya.

## Methods

### Study setting and population

The current study is an *a priori* exploratory secondary analysis of data from a prospective evaluation of the delivery of standard HTS to injured persons seeking emergency care at Kenyatta National Hospital (KNH) [18]. KNH, located in Nairobi, is the largest public health centre in Kenya. The facility maintains an A&ED (Accident and Emergency Department) that provides uninterrupted care access and specialty services. Within the KNH A&ED, clinical staff dedicated to Voluntary Counselling and Testing (VCT) services for HIV offer free HTS at all times. HTS at KNH is supported by the Kenyan MOH and follow national reporting procedures and linkage to care, through which anyone diagnosed with HIV is offered follow-up treatment services at a national comprehensive care centre [20].

### Study design

All adult patients (>8 years of age) who presented for A&ED injury care to KNH were eligible for recruitment. Enrolment was carried out between 1 March and 25 May 2021. Patients known to be pregnant, prisoners of the state, and those unable to provide informed consent were excluded. Additionally, participants lacking psychometric Likert data from the study survey were excluded from this analysis. Injury designation was based on the standardised triage classification used in the study setting, by clinical staff who were independent of the research personnel [21]. The Alcohol Use Disorders Identification Test-Concise (AUDIT-C) tool was used to evaluate alcohol use in data collection. The study was approved by the KNH ethics and research committee (P29/01/2020) and the Rhode Island Hospital Institutional Review Board (1501033-3). The Strengthening the Reporting of Observational Studies in Epidemiology guidelines were followed [22].

### Data collection

Trained study personnel were present in the A&ED 24 h a day during the enrolment period to screen, consent, and collect standardised data using digital databases [23]. Data included information on sociodemographic characteristics, past medical history, previous health behaviours pertaining to HIV testing and prevention, and use of alcohol (independent predictor variables); and psychometric Likert data on ED-based HIVST programming (dependent variables). The psychometric questionnaire was developed based on the Health Promotion Model (HPM). A HPM was utilised as it frames understanding of attitudes on health-related decisions by considering lifestyle, psychological health, and socio-cultural environment, which represent factors with contextual importance around HTS and HIVST [24]. The psychometric questionnaire was comprised of Likert items with a five-point scale representing levels of agreement: ‘do not agree at all’, ‘agree a little’, ‘agree somewhat’, ‘agree a lot’, and ‘agree completely’. The Likert items were informed by prior literature on HIVST [25,26], translated into Kiswahili, and piloted for understandability and reproducibility in the study setting. The 11 Likert items assessed three domains of acceptability for ED-based HIVST delivery: 1) acceptability of HIVST distribution in the A&ED (‘general acceptability’, three items), 2) acceptability for personal use of HIVST from the A&ED (‘personal acceptability’, four items), and 3) acceptability of HIVST distribution to/from social or sexual network members (‘distribution acceptability’, four items) (Supplement 1).

### Statistical analysis

Data analysis was completed using STATA version 16.0 (College Station, USA). The primary objective in the current analysis was to assess for factors associated with acceptability for ED-based HIVST programming across the three pre-defined domains. Characteristics of the study population were described and summarised, and the Likert item data were summarised nonparametrically using medians and inter-quartile ranges (IQRs). The component Likert items were aggregated to create a single outcome metric for each of the three domains of interest for which they comprised (Supplement 1). Component Likert items and corresponding scale responses were assessed for significant differences using Kruskal–Wallis tests to evaluate for intra-domain validity of the component items. Response distributions for the Likert data were analysed and presented with frequency proportions by item and domains. Ordinal logistic regression models were run for each of the three scale domains to yield unadjusted odds ratios with associated 95% confidence intervals (CIs) and p-values. The ordinal regressions were used to quantify the magnitude of the effect on odds of change in agreement across the five-point response scale based on predictors of interest (reference group “do not agree at all). Multivariate ordinal logistic regression models yielding adjusted odds ratios (aOR) were created for each of the three domains. Predictor variables were chosen a *priori* based on prior literature [27–30] and based on univariate analysis for those variables with *p* values <0.05. Multivariate model fitness was assessed using iterative likelihood ratio testing with variable addition (data not shown). Covariate predictor variables included sex, age, relationship status, educational attainment, previous HIV testing, HIV testing in the last 6 months, established primary care provider (PCP), and reported alcohol use.

## Results

### Populations characteristics

**Figure 1.**
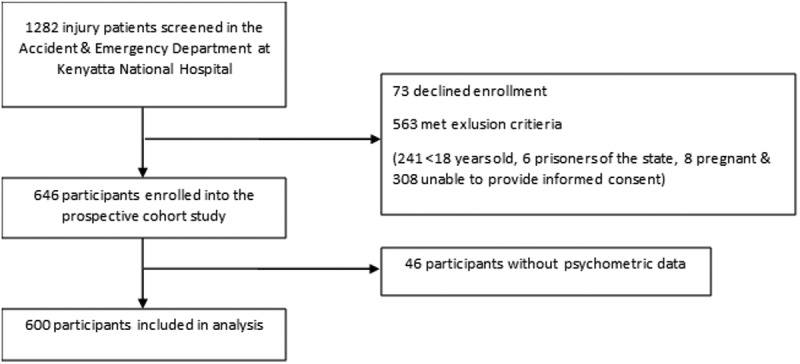
Study screening and enrollment flowchart.

Of 1,282 injury patients screened in the ED setting, 600 met inclusion for the *a priori* HIVST acceptability analysis (Figure 1). The majority of participants (88.7%) were male, with a median age of 29 years (IQR: 25, 37 years). Nearly one-half (46.4%) reported being married and just over half (55.0%) reported having an established PCP. While 86.2% had been tested for HIV at least once previously, 40.5% reported HIV testing within the preceding six months, with 1.8% of included participants self-identifying as PLHIV. The most common forms of injury were road traffic accidents (51.9%), and 42.1% had been transferred from another health facility. The median time from injury to presentation was 12.5 h (IQR: 5.0, 24.5 h) and the median time of ED care duration was 4.2 h (IQR: 2.5, 6.0 h) (Table 1).

**Table 1.** Population demographics, health characteristics, and risk factors.

Variable	n (%) or Median (IQR)
*Sex*	
Male	532 (88.7%)
Female	68 (11.3%)
*Age (years)*	29 (25, 37)
*Employment Status*	
Not working	107 (17.8%)
Laborer	303 (50.5%)
Self-employed	120 (20.0%)
Professional	70 (11.7%)
*Relationship Status*	
Single	200 (33.5%)
Married	277 (46.4%)
In a relationship	73 (12.2%)
Divorced/Widowed	42 (7.0%)
Wishes not to disclose	4 (0.7%)
Missing/Unknown	4 (0.7%)
*Education*	
Completed primary schooling (or less)	232 (38.7%)
Completed secondary schooling	248 (41.3%)
Greater than secondary schooling	120 (20.0%)
*Reported Chronic Medical Condition*	
Yes No	32 (5.3%) 568 (94.7%)
*Has Primary Care Provider*	
Yes	330 (55.0%)
No	270 (45.0%)
*Uses Recreational Alcohol*	
Yes	296 (49.3%)
No	297 (49.5%)
Missing/Unknown	7 (1.2%)
*Frequency of Condom Use*	
Always	110 (18.3%)
Sometimes	122 (20.3%)
Never	311 (51.8%)
Wishes not to disclose	13 (2.2%)
Missing	44 (7.3%)
*Previously Tested for HIV*	
Yes	517 (86.2%)
No	80 (13.3%)
Wishes not to disclose	1 (0.2%)
Missing	2 (0.3%)
*Timing of Last HIV Testing*	
≤ 6 months prior	243 (40.5%)
> 6 months prior	274 (53.0%)
Missing	83 (13.8%)
*Prior HIV Test Results*	
Positive	11 (1.8%)
Negative	488 (81.3%)
Wishes not to disclose	6 (1.0%)
Missing	95 (15.8%)

### HIV self-test acceptability

**Figure 2.**
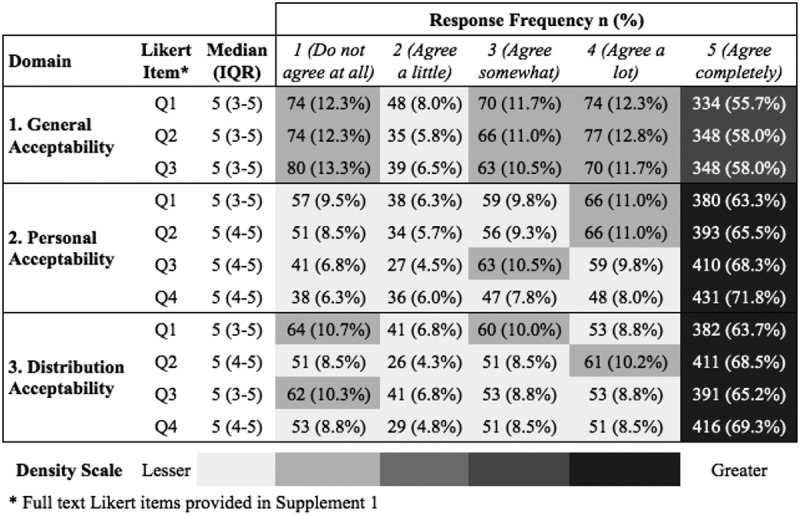
Response distributions stratified by domain and likert item.

Figure 2 illustrates the response distributions for each item within the three domains of interest. For Domain 1 (General Acceptability) the median response value on the five-point scale was 5 (IQR 2,5), and an average of 57.2% of the participants selected ‘Agree Completely’ for each of the three component items, with 55.7% for Q1, 58.0% for Q2 and 58.0% for Q3. This domain had the greatest variance of the three domains, with greater than 10% responding ‘Do not agree at all’ for each of the three questions (Figure 2). Unadjusted ordinal regression outputs are shown in Table 2. The multivariable analysis found that there was a higher odds of agreement with acceptability of general HIVST delivery from the ED for those who had previously been tested for HIV (aOR = 1.67 95% CI: 1.27–2.21), those who were 25 years and younger (aOR = 1.68 CI: 95% 1.36–2.08) and those who had a PCP (aOR = 1.24 95% CI: 1.03–1.52). Conversely, those who had a relationship status of single had a lower likelihood of responding with favourable general acceptability for HIVST delivery from the ED for (aOR = 0.79 95% CI: 0.65–0.96) (Table 3).

**Table 2.** Ordinal regression by topical domain.

	Domain 1: General Acceptability		Domain 2: Personal Acceptability		Domain 3: Distribution Acceptability	
Variable	OR (95% CI)	p-value	OR (95% CI)	p-value	OR (95% CI)	p-value
Male^1^	0.89 (0.66–1.20)	0.45	1.47 (1.12–1.93)	0.006	1.32 (1.00–1.73)	0.047
< = 25 Years^2^	1.68 (1.36–2.08)	<0.001	1.07 (0.88–1.31)	0.50	1.08 (0.89–1.32)	0.44
Not in Relationship^3^	0.79 (0.65–0.96)	0.019	1.21 (1.00–1.45)	0.051	1.05 (0.88–1.27)	0.58
Secondary School Education^4^	1.04 (0.84–1.28)	0.72	0.76 (0.62–0.93)	0.007	0.8 (0.66–0.97)	0.025
Post-Secondary School Education^4^	1.06 (0.81–1.37)	0.68	0.94 (0.73–1.20)	0.61	1.03 (0.81–1.33)	0.78
Previously HIV tested^5^	1.67 (1.27–2.21)	<0.001	1.83 (1.41–2.38)	<0.001	1.79 (1.38–2.33)	<0.001
Not HIV Tested in Last 6 Months^6^	0.95 (0.78–1.16)	0.62	0.97 (0.78–1.18)	0.76	1.15 (0.95–1.38)	0.16
Has a Primary Care Provider^7^	1.24 (1.03–1.52)	0.023	3.31 (2.72–4.03)	<0.001	2.42 (2.01–2.92)	<0.001
Drinks Alcohol^8^	1.17 (0.97–1.41)	0.094	0.81 (0.68–0.97)	0.019^a^	0.82 (0.69–0.98)	0.027

^a^Reference group: Female.

^b^Reference group: Greater than 25 Years.

^c^Reference group: In Relationship.

^d^Reference group: Primary School Education.

^e^Reference group: Not Previously Tested for HIV.

^f^Reference group: Tested for HIV in the last 6 Months.

^g^Reference group: Does not have a Primary Care Provider.

^h^Reference group: Does not Drink Alcohol.

**Table 3.** Multivariable regression by topical domain.

	Domain 1: General Acceptability		Domain 2: Personal Acceptability		Domain 3: Distribution Acceptability	
Variable	AOR (95% CI)	*p*-value	AOR (95% CI)	*p*-value	AOR (95% CI)	*p*-value
Male^a^	0.91 (0.69–1.22)	0.54	1.10 (0.86–1.43)	0.45	1.14 (0.88–1.47)	0.33
< = 25 Years^b^	1.42 (1.16–1.73)	0.001	0.95 (0.80–1.14)	0.60	1.00 (0.83–1.19)	0.97
Not in Relationship^c^	0.81 (0.67–0.97)	0.022	0.98 (0.82–1.16)	0.78	0.93 (0.79–1.10)	0.42
Secondary School Education^d^	1.12 (0.92–1.36)	0.027	0.80 (0.66–0.96)	0.018	0.81 (0.68–0.98)	0.029
Post-Secondary School Education^d^	1.21 (0.95–1.56)	0.13	1.07 (0.85–1.36)	0.56	1.11 (0.88–1.41)	0.034
Previously HIV tested^e^	1.68 (1.31–2.17)	<0.001	2.00 (1.59–2.53)	<0.001	1.81 (1.43–2.30)	<0.001
Not HIV Tested in Last 6 Months^f^	0.81 (0.67–0.97)	0.023	0.75 (0.63–0.89)	0.001	0.89 (0.76–1.06)	0.20
Has A Primary Care Provider^g^	1.39 (1.16–1.67)	<0.001	3.32 (2.76–4.00)	<0.001	2.45 (2.05–2.93)	<0.001
Drinks Alcohol^h^	1.13 (0.94–1.35)	0.20	0.85 (0.72–1.00)	0.052	0.84 (0.72–1.00)	0.047

^a^Reference group: Female.

^b^Reference group: Greater than 25 years.

^c^Reference group: In Relationship.

^d^Reference group: Primary School Education.

^e^Reference group: Not Previously Tested for HIV.

^f^Reference group: Tested for HIV in the last 6 months.

^g^Reference group: Does not have a Primary Care Provider.

^h^Reference group: Does not Drink Alcohol.

For Domain 2 (Personal Acceptability) the median response value on the five-point scale was 5 (IQR 3,5), and an average of 67.2% of participants selected ‘Agree Completely’ with each Likert item, with 63.3% for Q1, 65.5% for Q2, 68.3% for Q3 and 71.8% for Q4. The only response distributions above 10%, other than for ‘Agree Completely’, were for ‘Agree a Lot’ for Q1 and Q2 and ‘Agree Somewhat’ for Q3. In the multivariable analysis, there was a higher odds of reported agreement with acceptability of HIVST delivery from the ED for self-use among those who had previously been tested for HIV (aOR = 1.83 95% CI: 1.41–2.38), males (aOR = 1.47 95% CI: 1.12–1.93), and those with a PCP (aOR = 3.31 95% CI: 2.72–4.03). For those who reported alcohol use (aOR = 0.81 95% CI: 0.68–0.97) and those with a secondary school education (aOR = 0.76 95% CI: 0.62–0.93) there was a lower likelihood of participants acceptability agreement with HIVST delivery from the ED for self-use (Table 3).

For Domain 3 (Distribution acceptability to/from social or sexual networks) the median response value on the five-point scale was 5 (IQR 3,5), and an average of 66.0% of the participants ‘Agree Completely’ with each item (63.7% for Q1, 68.5% for Q2, 65.2% for Q3 and 69.3% for Q4). Over 10% of the participants responded ‘Do not Agree at all’ for Q1 and Q3 (Figure 2). There was a higher odds of reporting a favourable agreement response for Distribution Acceptability in participants who had previously been tested for HIV (aOR = 1.79 95% CI: 1.38–2.33), males (aOR = 1.32 95% CI: 1.00–1.73) and those with a PCP (aOR = 2.42 95% CI: 2.01–2.92). Among participants that reported alcohol use, there was a reduced likelihood for reporting favourable acceptability for HIVST delivery from the ED for network distribution and use (aOR = 0.82 95% CI: 0.69–0.97). A reduced odds of favourable agreement were also present among those with a secondary school education (aOR = 0.80 95% CI: 0.66–0.97) (Table 3).

## Discussion

Progress has been made in controlling the HIV epidemic; however, there exist persistent inequities in HTS in Africa [7]. HIVST programming from emergency care settings may represent an approach to help address this challenge. In the current analysis across all domains and their component items, the majority of participants responded favourably to HIVST availability in the ED setting. These data suggest that emergency care patients in Kenya would be receptive to HIVST access, and such programming may serve to increase HTS and subsequent identification of PLHIV, particularly among young people, and men specifically, who prioritize populations within the Kenya AIDS Strategic Framework to enhanced HIV care [8]. However, additional research involving healthcare provider perspectives and systems level evaluation are needed to inform the feasibility of HIVST implementation.

Although most patient respondents reported the highest level of agreement for HIVST acceptability, there were identified factors significantly associated with changes in the likelihood of agreement, representing potential barriers or facilitators to acceptability. Specifically, greater ED-HIVST acceptability was observed across all three domains for those who reported having a PCP and those that had been previously tested for HIV. As well, there were domain-specific predictors for increased likelihood of reported acceptability, which included for General Acceptability (Domain 1) being younger and for Personal Acceptability (Domain 2) being male. These data indicate that patients in ED settings who are more engaged in health care may be more likely to favourably perceive ED-HIVST, as well as those that have been shown to test less frequently and account for incident cases in Kenya, men and younger adults, respectively [2]. The impact of these findings is two-fold. For those who are less engaged with primary care and existing HIV services, especially young men, there is room for increased education about the risk for HIV and the importance of regular HIV testing [32,33], and increased accessibility of HIVST as a convenient and destigmatizing option for HIV testing [34]. For those who engage in HIVST this mechanism could help meet Kenyan national HIV testing goals and aligns with the Kenya AIDS Strategic Framework which identifies self-testing as a target intervention and component of prevention for younger men who, compared to women, have a delayed time to diagnosis for HIV [8,20].

While existing data on the acceptability of HIVST in African ED settings are limited, HIVST has been shown to be a successful mechanism to testing among populations not well engaged in conventional programming and is recommended by the WHO [6]. In the present data approximately one in every eight respondents reported never before testing for HIV, which is consistent with predominately male populations known to be disengaged with standard HTS as compared to females [2]. As the current analysis indicates that HIVST is perceived as acceptable in the emergency care population, there is potential to increase testing if implemented among these demographic groups that have been historically difficult to access. Previous work has also suggested expanding HIVST distribution by allowing individuals to distribute HIVST kits to social and/or sexual networks which could magnify distribution and testing impacts [11]. As the current results show that the distribution of HIVST from the ED for use within social or sexual networks was acceptable there is potential for increased testing impacts, however further research is needed to evaluate approaches that will be most appropriate to do so. As emergency care is a complex practice arena such work would likely best be achieved via mixed methods assessments that study all relevant stakeholders inclusive of patients, policymakers and healthcare personnel.

Additionally, while the current data points to high potential acceptability of HIVST among emergency care populations in Kenya, other studies of HIVST in African settings have reported barriers to success such as cost, user error and linkage to treatment [35,36]. The current study did not examine the reasons for perceived acceptability or specific barriers from the emergency care setting; therefore, evaluation of these aspects via implementation science methods will be key in any ED-based HIVST programming to ensure best practices regarding target populations, logistics of distribution, mechanisms for confirmatory testing and linkage to care.

HIVST programming has the potential to offload human resources, mitigate distribution access inequities, lower stigmatizing experiences, and increase the number of tests performed [35,36]. While the COVID-19 pandemic continues and individuals are presenting for emergency care for injury and health concern, the ongoing HIV epidemic must not be neglected [31,32,37]. Our study suggests that ED-HIVST may be an acceptable approach to reduce resource needs for HTS and help identify undiagnosed HIV in populations as they seek emergency care, particularly when unable to access other testing services due to pandemic-related restrictions and treatment limitations.

## Limitations

The current exploratory analysis does have limitations. The study took place in a large, urban health centre in Kenya, where HIV counselling and care are relatively accessible to those who engage in HTS. Given this, the findings may not be generalisable to less well-resourced settings, however as HIVST empowers individual test recipients, the resource barriers can be overcome with self-test programming. Additionally, at ~5% Nairobi has a lower prevalence of HIV than some other regions in Kenya, which could impact acceptability for HIVST due to differential risk profiles. Also, the lack of inclusion of persons less than 18 years of age limits application to the younger spectrum of the adolescent demographic. The Likert scale may have allowed for acquiescence bias or habituation bias, but the consistency in response distributions within domains for the component Likert items in conjunction with the large sample size make this less likely [38]. There were some missing psychometric data from the primary study population, which could have introduced error; however, the missingness was minimal (<8%) and is therefore not likely to substantially impact the findings. Additionally, although ordinal analyses provide information on trends within the response scales utilised, the nature of the statistical approach does not allow the identification shifts in response likelihoods between individual levels of agreement, which could be important for the assessments with more divergent response distributions to better understand aspects of perceived acceptability for ED-based HIVST delivery. Finally, while current data report on perceived acceptability, additional research is needed assessing patient uptake of HIVST in emergency care settings, as well as perceived barriers and facilitators to implementation of HIVST.

## Conclusion

The current exploratory data indicate high acceptability by patients for potential uptake of HIVST in an ED setting in Kenya, particularly among patients with prior engagement in health care but also among those who have been identified as priority populations in epidemic control programming. The findings agree with WHO guidelines recommending the delivery of HIVST to increase testing and identification of PLHIV and provide data that can be used to inform further research and programmatic design. Specifically, future research on HIVST in emergency care venues should consider population characteristics, as well as information on systems aspects, health practitioner perspectives, and patient uptake in order to inform implementation strategies.

## Supplementary Material

Supplemental Material

## Paper context

HIV self-test programming from emergency departments represents an innovative approach to address challenges in HIV testing services for high-risk and poorly engaged populations in Africa. In the current analysis of 600 patient participants from Kenya the majority viewed emergency department-based HIV self-test access as favourable for self-use and for distribution to contacts. These data suggest that Kenyan emergency care patients are receptive to HIV self-testing access, and such programming may serve to increase testing.
