# Supplementary material for: HIV self-testing acceptability among injured persons seeking emergency care in Nairobi, Kenya
Source: Glob Health Action. 2023 Jan 11;16(1):2157540. doi: 10.1080/16549716.2022.2157540 (PMC9848354; doi:10.1080/16549716.2022.2157540)
Supplement: Supplemental Material [file ZGHA_A_2157540_SM1788.docx]

**Supplements**

Supplement 1: Likert Items and Topical Focus

| **Likert Scale Domains** | **Likert Item*** | **Topical Focus** |
| --- | --- | --- |
| Domain 1:  General acceptability | Q1: The Accident and Emergency Department should provide an    option for patients to complete HIV self-tests while they are being    cared for in the Accident and Emergency Department | The Emergency Department should provide HIVST for immediate use |
|  | Q2: The Accident and Emergency Department should provide an option for patients to complete HIV self-tests and be counselled by medical providers after they take their HIV self-tests while they are still in the Accident and Emergency Department | The Emergency Department should provide HIVST for immediate use and counseling |
|  | Q3: The Accident and Emergency Department should provide an option for patients to be given a HIV self-test kit to be used outside of the Accident and Emergency Department by the patient themselves | The Emergency Department should provide HIVST for outside use |
| Domain 2:  Personal acceptability | Q1: If an HIV self-test kit was offered in the Accident and Emergency Department to be used by you while you were in the Accident and Emergency Department, you would accept the testing | The respondent would accept HIVST for immediate use |
|  | Q2: If an HIV self-test kit was offered in the Accident and Emergency Department to be used by you after you left the Accident and Emergency Department, you would accept the testing kit | The respondent would accept HIVST for outside use |
|  | Q3: If you were given an HIV self-test kit from the Accident and Emergency Department you would want to be contacted to help obtain additional medical care if needed after using the HIV self-test kit | The respondent would accept HIVST and counseling |
|  | Q4: If you used an HIV self-test and the results were positive for HIV, meaning you might be infected with HIV, you would seek additional HIV care and treatments | Willingness to seek care post-testing |
| Domain 3: Distribution acceptability  to/from  social  or sexual networks | Q1: You would be willing to give an HIV self-test kit to a friend or social contact for them to test themselves if you received an HIV self-test kit from the Accident and Emergency Department | Willingness to distribute HIVST kit to friends |
|  | Q2: You would be willing to give an HIV self-test kit to a sex partner to help them get tested if you received an HIV self-test kit from the Accident and Emergency Department | Willingness to distribute HIVST kit to sex partners |
|  | Q3: You would be willing to accept an HIV self-test kit from a friend or social contact to test yourself if they received an HIV self-test kit from the Accident and Emergency Department | Willingness to accept HIVST kit from social contact |
|  | Q4: You would be willing to accept an HIV self-test kit from a sex partner to test yourself if they received an HIV self-test kit from the Accident and Emergency Department | Willingness to accept HIVST from sex partner |

*The five-point Likert scale included the following options: “do not agree at all”, “agree a little”, “agree somewhat”, “agree a lot”, and “agree completely”.
